# Cause rare de baisse d'acuité visuelle chez la femme enceinte

**DOI:** 10.11604/pamj.2014.17.86.3913

**Published:** 2014-02-03

**Authors:** Zouheir Hafidi, Rajae Daoudi

**Affiliations:** 1 Université Mohammed V Souissi, Service d'Ophtalmologie A de l'Hôpital des Spécialités, Centre Hospitalier Universitaire, Rabat, Maroc

**Keywords:** Acuité visuelle, fond d’œil, choriorétinopathie séreuse, visual acuity, fundus, serous chorioretinopathy

## Image en medicine

Une patiente âgée de 28 ans est admise pour une baisse brutale d'acuité visuelle de l'oeil gauche. A l'interrogatoire on note qu'elle est enceinte de 4 mois. L'examen ophtalmologique retrouve une acuité visuelle à 2/10e au niveau de l'oeil gauche. Au fond d'oeil: un soulèvement rétinien occupant la région maculaire de l'oeil gauche avec des lésions jaunâtres disséminées au pôle postérieur des deux yeux. L'examen général n'a pas montré d'anomalies (Tension Artérielle: 13/08 cmhg). L'angiographie à la fluorescéine a mis en évidence un point de fuite unique avec remplissage progressif de la bulle du décollement, et de multiples taches hyper fluorescentes correspondant à des altérations de l’épithélium pigmentaire. Le diagnostic d'une choriorétinopathie séreuse centrale (CRSC) a été retenu. L’évolution a été spontanément favorable après l'accouchement. La choriorétinopathie séreuse centrale est une affection rétinienne essentiellement du sujet jeune. Sa physiopathogénie fait intervenir une altération diffuse de la fonction pompe de l’épithélium pigmentaire entrainant une accumulation de liquide sous la rétine à l'origine du DSR. Ce dysfonctionnement peut être d'origine vasculaire (ex: Hyper Tension Artérielle) ou toxique (ex: rôle déclenchant et ou aggravant des corticostéroïdes). Les facteurs de risque reconnus sont le sexe masculin, le stress psychologique (taux élevés de stéroïdes endogènes), l'administration d'une corticothérapie, une Hyper Tension Artérielle incontrôlée, certaines pathologies endocrines (Syndrome de Cushing ou tumeurs sécrétrices d'hormones stéroïdes), ainsi que la grossesse (souvent apparition du DSR au cours du 3ème trimestre avec une disparition spontanée en fin de grossesse ou peu après l'accouchement. Une nouvelle poussée peut survenir lors d'une nouvelle grossesse).

**Figure 1:**
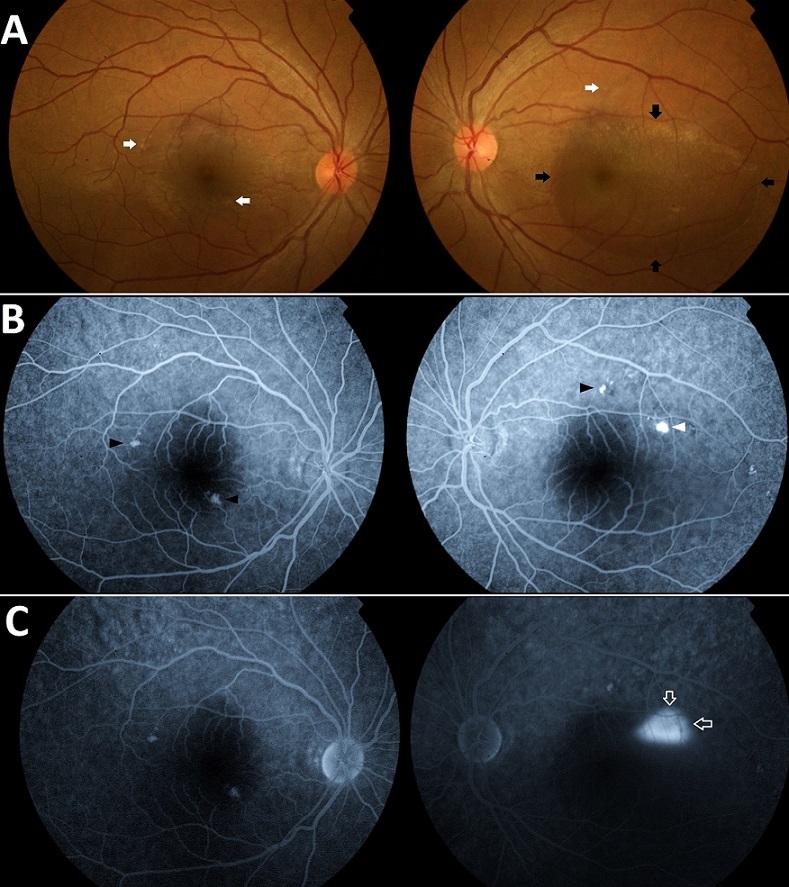
A) Aspect du fond d’œil à l'admission montrant un soulèvement rétinien à limites nettes au niveau de la région maculaire de l’œil gauche (flèches noires) d'environ 6 diamètres papillaires de diamètre, avec des lésions jaunâtres profondes bilatérales correspondant à des altérations de l’épithélium pigmentaire (flèches blanches); B) Phases précoces de l'angiographie à la fluorescéine mettant en évidence un point de fuite para maculaire temporal supérieur au niveau de l’œil gauche (tête de flèche blanche). On note aussi un effet fenêtre des lésions jaunâtres constatées au fond d’œil (tête de flèches noires). C)Temps tardif de l'angiographie à la fluorescéine montrant un remplissage progressif de la bulle de DSR en tache d'huile (flèches blanches)

